# Controlled dietary phosphate loading in healthy young men elevates plasma phosphate and FGF23 levels

**DOI:** 10.1007/s00424-024-03046-4

**Published:** 2024-11-27

**Authors:** Jennifer Scotti Gerber, Eva Maria Pastor Arroyo, Johanne Pastor, Miguel Correia, Stefan Rudloff, Orson W. Moe, Daniela Egli-Spichtig, Nilufar Mohebbi, Carsten A. Wagner

**Affiliations:** 1 Division of Nephrology, University Hospital Zurich, Zurich, Switzerland; 2 Division of Nephrology, Ente Ospedaliero Cantonale, Lugano, Switzerland; 3 Institute of Physiology, University of Zurich, Winterthurerstrasse 190, CH-8057 Zurich, Switzerland; 4 Charles and Jane Pak Center for Mineral Metabolism and Clinical Research, University of Texas Southwestern Medical Center, Dallas, TX 75390 USA; 5 Division of Nephrology and Hypertension, University of Bern and University Hospital Bern, Bern, Switzerland; 6 Department of Internal Medicine, Division of Nephrology, University of Texas Southwestern Medical Center, Dallas, TX 75390 USA; 7 Department of Physiology, University of Texas Southwestern Medical Center, Dallas, TX 75390 USA; 8 National Center of Competence in Research, NCCR Kidney.CH, Zurich, Switzerland

**Keywords:** Diet, Phosphate, FGF23, Human trial, Health risk

## Abstract

Increased dietary inorganic phosphate (P_i_) intake stimulates renal P_i_ excretion, in part, by parathyroid hormone (PTH), fibroblast growth factor 23 (FGF23) or dopamine. High dietary P_i_ may also stimulate sympathetic outflow. Rodent studies provided evidence for these regulatory loops, while controlled experiments in healthy humans examined periods of either a few hours or several weeks, and often varied dietary calcium intake. The effects of controlled, isolated changes in dietary P_i_ intake over shorter periods are unknown. We studied the effects of a low or high P_i_ diet on parameters of mineral metabolism in 10 healthy young men. Participants received a standardized diet (1000 mg phosphorus equivalent/day) supplemented with either a phosphate binder (low P_i_ diet) or phosphate capsules (750 mg phosphorus, high P_i_ diet) in a randomized cross-over trial for 5 days with a 7-day washout between diets. High P_i_ intake increased plasma P_i_ levels and 24-h excretion and decreased urinary calcium excretion. High P_i_ intake increased intact FGF23 (iFGF23) and suppressed plasma Klotho without affecting cFGF23, PTH, calcidiol, calcitriol, Fetuin-A, dopamine, epinephrine, norepinephrine, metanephrine, or aldosterone. Higher iFGF23 correlated with lower calcitriol and higher PTH. These data support a role for iFGF23 in increasing renal P_i_ excretion and reducing calcitriol in healthy young men during steady-state high dietary P_i_ intake. High dietary P_i_ intake elevated blood P_i_ levels in healthy young subjects with normal renal function and may therefore be a health risk, as higher serum P_i_ levels are associated with cardiovascular risk in the general population.

**Supplementary Information:**

The online version contains supplementary material available at 10.1007/s00424-024-03046-4.

## Introduction

Phosphate (P_i_) is an essential mineral required for many critical processes such as structural part of cellular membranes with phospholipids, storing genetic information in DNA and RNAs, cellular energy metabolism (ATP), signaling (phosphorylation, GTP), muscle contraction and relaxation, as buffer for H^+^, and to provide structural integrity to bone in the form of apatite [1]. P_i_ deficiency causes muscle weakness, anemia, insulin resistance, acidosis, and rickets or osteopenia while P_i_ excess can result in ectopic calcifications and is associated with increased cardiovascular morbidity and mortality that is particularly pronounced in patients with reduced kidney function. Systemic P_i_ homeostasis is achieved by the balance between intestinal P_i_ absorption, deposition in and release of P_i_ from bone and soft tissues, and renal excretion [37]. Intestinal P_i_ absorption proceeds via active transcellular transport and passive paracellular pathways [26]. Transcellular transport operates mostly under conditions of low P_i_ availability and is highly regulated. Under conditions of high dietary P_i_, P_i_ is predominately absorbed through the paracellular route which appears not to adapt to P_i_ intake or systemic requirements. Under these conditions of largely ungated entry, the kidneys are the controllers of systemic P_i_ homeostasis by adapting the rate of tubular reabsorption of filtered P_i_ and thereby urinary P_i_ excretion. Tubular reabsorption of P_i_ is mediated by at least three distinct phosphate transporters, NaPi-IIa (*SLC34A1*), NaPi-IIc (*SLC34A3*), and Pit2 (*SLC20A2*) [11, 37, 60]. Inactivating genetic variants in *SLC34A1* and *SLC34A3* demonstrate the relevance of these transporters for renal and systemic P_i_ balance in humans [36]. The activity of NaPi-IIa and NaPi-IIc is highly regulated by a variety of hormones and factors that include parathyroid hormone (PTH), fibroblast growth factor 23 (FGF23), dopamine, calcitriol, glucocorticoids, growth hormone, potassium, or acid–base status [3, 11, 33, 37]. Again, human genetics have demonstrated the importance of PTH and FGF23 for systemic P_i_ balance [30].

In industrialized countries, estimated dietary intake of P_i_ is high and exceeds recommended daily allowance due to high consumption of animal protein (organic phosphate) and processed food frequently containing food additives (highly bioavailable inorganic phosphate) [7, 8, 56]. High dietary P_i_ intake has been associated with higher hazard ratios for cardiovascular disease and mortality in the general population [9, 15, 16]. However, it has been debated whether prolonged high dietary P_i_ intake could cause higher serum P_i_ levels in subjects with normal kidney function raising the question of whether serum P_i_ is truly independent of dietary P_i_ or is this due to limitation of sensitivity of detection methods.

The impact of changes in dietary P_i_ intake on endocrine regulators, renal, and intestinal P_i_ handling has been extensively studied in mice and rats [6, 26, 28, 37, 46]. In contrast, only a few studies addressed this topic in healthy humans while dysregulation in patients with end stage kidney disease (ESKD) has been thoroughly examined. An early study by Spencer et al. provided 7 patients with high and low P_i_ and calcium diets over periods of 12–42 days, demonstrating diet-dependent renal adaptation of P_i_ excretion [52]. In short-term studies, where healthy subjects were orally loaded with P_i_ and followed up for eight hours, a post-prandial rise in serum P_i_ and urinary P_i_ excretion was observed. This was accompanied by an increase in PTH and, in some studies, by an increase in intact FGF23 (iFGF23) [44, 59]. As part of the analysis of patients with chronic kidney disease (CKD), ESKD or after transplant, 6 healthy subjects were given 2 days of P_i_ restricted diet and 3 days with P_i_ repleted diet, but no changes in the C-terminal FGF23 (cFGF23) fragment were found [34]. In mixed sex, intermediate-term studies, oral, enteral, or intravenous P_i_ loading increased iFGF23 and urinary P_i_ excretion, while enteral and intravenous P_i_ loading additionally increased serum P_i_, and PTH, and decreased calcitriol [2, 20, 25, 50, 58]. In a long-term study, 20 young subjects of both sexes were given low vs high dietary P_i_ over a period of 11 weeks (partly supplemented also with high vitamin D_3_). Subjects had higher plasma P_i_, iFGF23, PTH and soluble α-Klotho (referred to as soluble Klotho) levels as well as higher urinary P_i_ levels with a high P_i_ diet [41]. While these studies demonstrated that iFGF23 is regulated by dietary P_i_ intake, the findings related to calcitriol or PTH varied between studies. Moreover, interpretation of data is complicated as many studies concomitantly changed P_i_ and calcium intake. Also, little information is available on more intermediate adaptations to pure P_i_ challenges when it is expected that acute adaptation has been achieved and before long-term compensatory mechanisms may be activated.

Here, we addressed two main questions in healthy volunteers: (1) what is the endocrine response to low vs high P_i_ intake after 5 days of controlled diet and (2) does a higher intake of P_i_ for 5 days elevate blood P_i_ levels in healthy subjects with normal renal function?

## Methods

### Proband recruitment

We recruited 10 healthy young men aged 20–40 years from the general population. All volunteers were screened for kidney function (eGFR > 90 ml/min/1.73 m^2^ CKD-EPI 2009, no albuminuria), body weight (BMI), and parameters of mineral metabolism within the normal range. In case of low calcidiol levels, participants were first substituted with calcidiol until reaching values within the normal range before the study. We also screened blood pressure, heart rate, Hb1Ac as well as plasma electrolyte values. We excluded subjects with diabetes, known kidney disease (eGFR < 90 ml/min/1.73 m^2^ CKD-EPI 2009, albuminuria), history of kidney stones, hypertension (systolic or diastolic blood pressure above 140/85 mm Hg), hypotension (systolic or diastolic blood pressure below 90/60 mm Hg), taking regular medication, with known allergies, with hyper- or hypoaldosteronism, with hyper- or hypothyroidism, adhering to special dietary requirements (including vegetarian and vegan diets), or participating in another parallel study.

### Study design

**Fig. 1:**
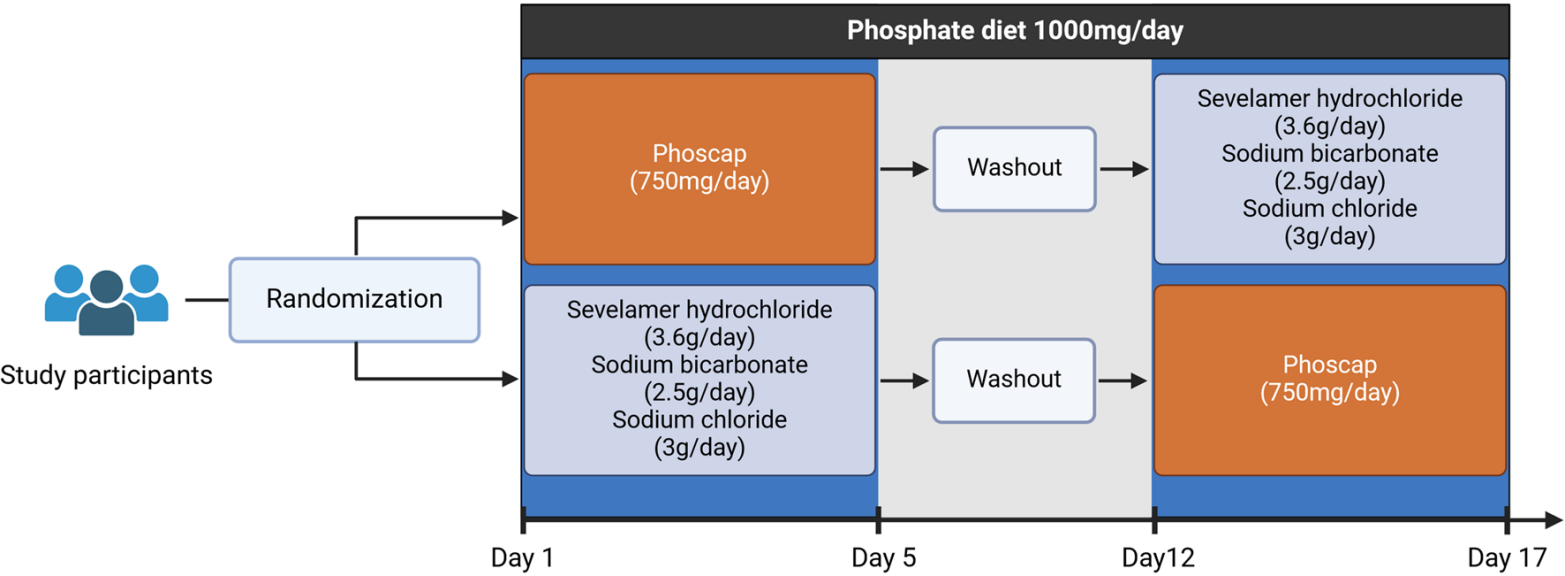
Experimental study design. Study participants received throughout the study a standardized P_i_ diet containing 1000 mg phosphorus per day. They were randomly assigned to either start with a 5-day period of additional phosphate capsules (Phoscap), corresponding to 750 mg of phosphorus per day (high P_i_ diet), or to receive 1200 mg of the phosphate binder sevelamer hydrochloride three times daily (low P_i_ diet). During the low P_i_ diet, participants were compensated for sodium intake with Phoscap with 500 mg of sodium bicarbonate 5 times a day and 1 g of sodium chloride three times a day. After a 7-day washout period, they switched to the opposite treatment for another 5-day period

This study was conducted as an open-label cross-over study. After screening, participants were randomized to either start with low or high P_i_ diet and then were switched after 7 days of wash-out to the opposite treatment (Fig. 1). All participants completed the trial.

All participants received standardized diets designed by a trained dietician that contained approximately 1000 mg phosphorus^1^/day during the entire phase of the study including the wash-out period between diets. The standardized menu and nutrition guidelines are documented in the Supplement. Participants were also instructed to avoid beverages or snacks containing high P_i_. In the low P_i_ arm, participants received the normal P_i_ diet supplemented with the phosphate binder sevelamer hydrochloride 1200 mg thrice daily for 5 days before each meal while in the high P_i_ arm, participants received the normal P_i_ diet supplemented with a total of 8 capsules of Phoscap (Dr. Bichsel AG, Interlaken, Switzerland) per day containing each 2.54 mmol Na_2_HPO_4_ and 0.48 mmol NaH_2_PO_4_ corresponding to 750 mg phosphorus per day for 5 days. Four Phoscap capsules were taken before breakfast and another 4 before dinner. To compensate for the sodium intake with Phoscaps, participants in the low P_i_ diet arm received Na/NaHCO_3_ supplements (Nephrotrans capsules, 500 mg 5 times per day and sodium chloride Galepharm 1 g 3 times per day). One Nephrotrans capsule was taken before each meal and 2 were taken in between the meals. One sodium chloride tablet was taken before each meal. This led by mistake to an overcompensation of sodium by 0.8 g (35 mmol) in the low P_i_ diet arm.

Twenty-four-hour urine was collected on day 5 of each dietary period. Thymol was added to urine collectors to reduce bacterial growth. Additionally spot urine was collected from the second morning urine at the end of day 5. The blood was collected at the end of each dietary period in the morning from overnight fasted participants.

### Biochemical blood and urine analyses

Blood and urine parameters were determined at the Department of Clinical Chemistry at the University Hospital Zurich using routine procedures. Tubular maximum P_i_ reabsorption (TmP/GFR) was calculated by using the fractional tubular reabsorption (TRP) of P_i_ with the following equations using spot urine [45]:

$$\begin{array}{l}TRP=1-\left[\left(\frac{{U}_{Pi}}{{P}_{Pi}}\right)*\left(\frac{{P}_{Cr}}{{U}_{Cr}}\right)\right]\\ TRP \le 0.86 \to TmP/GFR=TRP*{P}_{Pi}\\ TRP>0.86 \to TmP/GFR=\left(0.3*\frac{TRP}{1-\left(0.8*TRP\right)}\right)*{P}_{Pi}\end{array}$$

Daily fractional excretion of P_i_ (FEP_i_) was calculated by the following equation using the absolute 24-h urine P_i_ and creatinine:

$$FE{P}_{i}=\frac{{(U}_{Pi}*{P}_{Cr})}{{(P}_{Pi}*{U}_{Cr})}*100$$

Daily renal filtered P_i_ load was calculated with the following equation:

$$Renal\;P_iload/day=\left(\frac{U_{Cr}\ast U_{volume}}{P_{Cr}\ast1440}\right)\ast P_{Pi}$$

Plasma iFGF23, cFGF23, Fetuin-A, and urinary metanephrine were measured with the human iFGF23 and cFGF23 enzyme-linked immunosorbent assay (ELISA) (iFGF23 and cFGF23, Quidel, 60–6600 and 60–6100, respectively), Fetuin-A ELISA (R&D Systems, DFTA00, Lot P413286), and the Metanephrine Urine ELISA (Demeditec Diagnostics GmbH, DEE8400) according to manufacturers’ protocols. For Fetuin-A, plasma was diluted 1:8000 in RD5-26 before being assayed according to manufacturers’ protocols. To calculate the iFGF23/cFGF23 ratio, cFGF23 values were converted from RU/ml to pg/ml by multiplying by 2 (1 RU/ml = 2 pg/ml) [14]. Plasma soluble Klotho determination was described previously [5]. Briefly, 50 μl of human plasma was diluted 1:10 in Krebs–Ringer HEPES buffer (KRHB) and incubated (overnight, 4 °C) with 0.25 μg of the KM2076 (KAL-K0603, Cosmo BIO USA) antibody. Pierce A/G magnetic beads (cat. # 88803, Thermo Scientific, USA) were washed with KRHB, and added to the diluted plasma (3 h, 4 °C). The beads were then washed with KRHB and Klotho was eluted by adding 50 μl of 1X LDS electrophoresis sample buffer (Thermo Scientific), heating at 100 °C for 3 min and subsequently cooled on ice. DTT was added to the supernatant to a final concentration of 100 mM for gel electrophoresis. Twenty microliters of protein sample was electrofractioned on an LDS 4–12% Bis–Tris gel, and transferred onto a nitrocellulose membrane at 4 °C. The membrane was blocked in 5% milk (1 h, room temperature) and then incubated overnight at 4 °C with a 1:750 dilution (0.33 μg/ml) of primary antibody KM2119 (KAL-K0604, Cosmo BIO USA) in antibody diluent (Dako #S3022). The membrane was washed in TBS-0.1% Tween and then incubated with a horseradish peroxidase linked anti-rat IgG antibody (NA935, GE Healthcare) diluted in milk/TBS-T for 1 h, followed by stringent washes with TBS-T containing 0.5% Tween. Membrane was incubated with SuperSignal ECL™ West Femto Maximum Sensitivity substrate (Thermo Scientific, USA) and imaged using the ChemiDoc MP system (Bio-Rad, USA). Band intensities were quantified against a standard of mouse Klotho using ImageLab software (Bio-Rad, USA).

### Ethics

The study was approved by the local ethics committee (Kantonale Ethikkommission Zürich) under the number KEK-ZH 2014–0566. The study adhered to Declaration of Helsinki, ICH-GCP, GEP, and the Swiss law on human studies. Written informed consent was obtained from all participants before inclusion into this study.

### Statistics

Data was analyzed using GraphPad Prism software version 10. Data in tables are presented as mean ± standard deviation (SD) and in figures as individual points and as difference (Δ) between high P_i_ and low P_i_ diet expressed as mean difference ± 95% confidence interval. Data were analyzed either using paired samples *t*-test, Wilcoxon test, or non-linear regression (*α* = 0.05). For non-linear regression, a straight line was fitted, and the ROUT method was applied for outlier removal with a maximum false discovery rate of *Q* = 1% [43]. The best fit value of the slopes was tested for deviation from 0 by the extra sum-of-squares *F* test.

## Results

### Study participants and study protocol

Baseline characteristics of participants are listed in Table 1. The intake of diets containing low or high P_i_ had no significant effects on body weight, estimated GFR, urine volume, serum albumin, or 24-h urinary creatinine excretion (Table 2).

**Table 1:** Baseline characteristics of study participants

Parameter	Mean ± SD (*n* = 10)
Age (yrs)	29.0 ± 3.3
Weight (kg)	80.4 ± 12.8
BMI (kg/m^2^)	24. 3 ± 3.1
Blood pressure (syst, mm Hg)	118.2 ± 8.8
Blood pressure (diast, mm Hg)	75.0 ± 7.2
Heart rate (bpm)	71.5 ± 17.3
Serum creatinine (mmol/l)	76.9 ± 12.1
eGFR CKD-EPI 2009 (ml/min/1.73 m^2^)	114.3 ± 14.5
Serum calcidiol after suppl (mg/l)	25.4 ± 5.7
HbA1c (%)	5.2 ± 0.2
Albuminuria (mg/d)	7.4 ± 5.4
Serum PTH (ng/l)	40.4 ± 7.2
Blood Na^+^ (mmol/l)	141.0 ± 2.1
Blood K^+^ (mmol/l)	3.9 ± 0.2

**Table 2:** Body weight, estimated glomerular filtration rate (eGFR), plasma albumin, urine volume and creatinine after 5 days of low- or high-phosphate (P_i_) diet. Data are presented as mean ± SD

Parameter	Low P_i_ diet Day 5 (*n* = 10)	High P_i_ diet Day 5 (*n* = 10)	*p*-value^1^
Body weight (kg)	80.0 ± 12.5	80.6 ± 12.4	0.119
eGFR CKD-EPI 2009 (ml/min/1.73 m^2^)	106.6 ± 18.2	109.6 ± 14.1	0.274
Serum albumin (g/l)	45.5 ± 2.8	44.7 ± 1.8	0.235
Urine volume (l/24 h)	2.2 ± 0.7	1.9 ± 0.6	0.206
Urine creatinine (mmol/24 h)	16.7 ± 2.0	15.8 ± 2.6	0.309

^1^Paired *t*-test

### Effects of dietary P_i_ on plasma and urine minerals

**Fig. 2:**
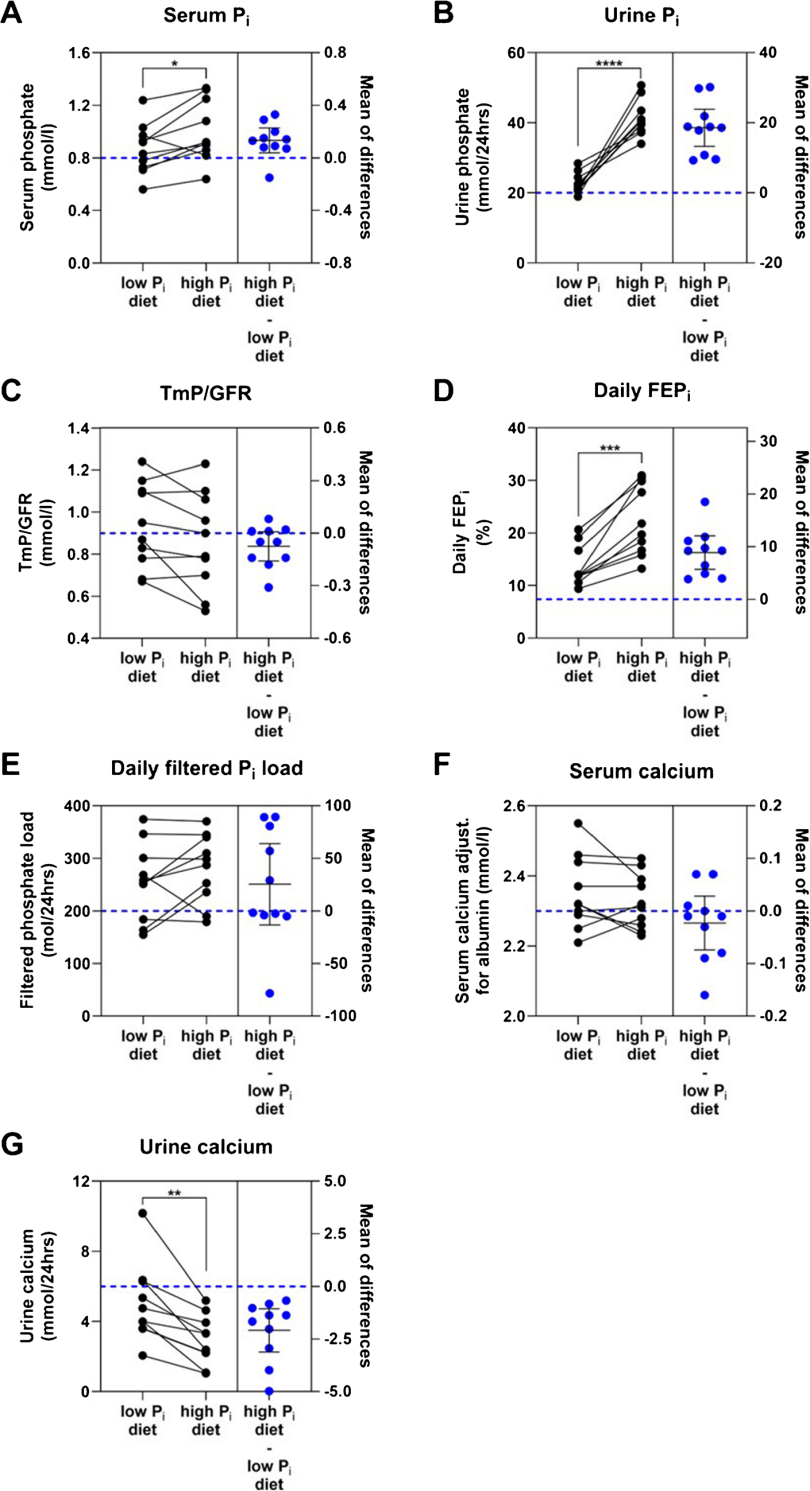
Serum and urine levels of phosphate (P_i_) and calcium and P_i_ reabsorption after 5 days of low or high P_i_ diet. Serum and 24-h urine P_i_ levels (**A**, **B**), tubular maximum P_i_ reabsorption (TmP/GFR) (**C**), fractional excretion (FEP_i_) (**D**), and filtered Pi load (**F**), and serum and 24-h urine calcium levels (**E**,** F**) on the final day of both the 5-day low and high P_i_ diet. Total plasma calcium was corrected for albumin. Blue dots represent differences (Δ) between high P_i_ diet and low P_i_ diet expressed as mean difference ± 95% confidence interval and blue dashed line represents the zero line of the right y-axis. Data was analyzed by the paired *t*-test, *n* = 10, *p < 0.05, **p < 0.01, ***p < 0.001, ****p < 0.0001

The study participants exhibited significantly lower levels of serum and 24-h urine P_i_ on day 5 of low dietary P_i_ intake compared to when they were on high dietary P_i_ intake (Fig. 2A and B, Table 3). TmP/GFR, a measure of renal tubular P_i_ reabsorption was not significantly different between the diets, however, showed in most participants a trend to decrease when they were on a high dietary P_i_ intake (Fig. 2C, Table 3). The daily FEP_i_ was increased, and the daily filtered P_i_ load was unchanged (Fig. 2D and E, Table 3).

**Table 3:** Serum and urine electrolytes after 5 days of low- or high-phosphate (P_i_) diet. Data are presented as mean ± SD

Parameter	Low P_i_ diet Day 5 (*n* = 10)	High P_i_ diet Day 5 (*n* = 10)	*p*-value^1^
Serum			
Sodium (mmol/l)	141.5 ± 2.8	141.2 ± 1.9	0.761
Potassium (mmol/l)	3.9 ± 0.2	3.9 ± 0.3	0.921
Chloride (mmol/l)	103.1 ± 2.7	103.2 ± 2.3	0.879
Phosphate (mmol/l)	0.87 ± 0.19	1.00 ± 0.23	**0.011**
Total calcium (mmol/l)	2.22 ± 0.07	2.21 ± 0.06	0.857
Urine			
Creatinine (mmol/24 h)	16.7 ± 2.0	15.8 ± 2.6	0.309
Sodium (mmol/24 h)	208.9 ± 63.8	174.0 ± 40.7	0.205
Potassium (mmol/24 h)	60.7 ± 15.3	67.3 ± 16.7	0.415
Chloride (mmol/24 h)	183.7 ± 52.5	128.2 ± 33.1	**0.028**
Phosphate (mmol/24 h)	22.7 ± 2.9	41.2 ± 5.1	** < 0.0001**
Calcium (mmol/24 h)	5.0 ± 2.2	2.9 ± 1.4	**0.0013**
pH	6.45 ± 0.6	6.25 ± 0.6	0.509
Calculated parameters			
Tmp/GFR (mmol/l)	0.94 ± 0.2	0.86 ± 0.2	0.074
Daily FEP_i_ (%)	13.6 ± 3.8	22.5 ± 6.7	**0.0001**
Daily filtered P_i_ load (mol/24 h)	255.4 ± 73.2	280.8 ± 65.1	0.171

^1^Paired *t*-test, *p* < 0.05 are bold

Dietary P_i_ intake had no effect on total serum calcium levels corrected for albumin (Fig. 2F, Table 3); however, 24-h urinary calcium excretion was significantly lower with high P_i_ intake (Fig. 2G, Table 3). Changes in dietary P_i_ intake had no effects on serum sodium, potassium, or chloride levels (Table 3). Likewise, 24-h urinary excretion of sodium and potassium as well as urine pH remained unaltered while low dietary P_i_ intake significantly increased 24-h urinary chloride excretion (Table 3).

### Effects of dietary P_i_ on phosphatropic hormones

**Fig. 3:**
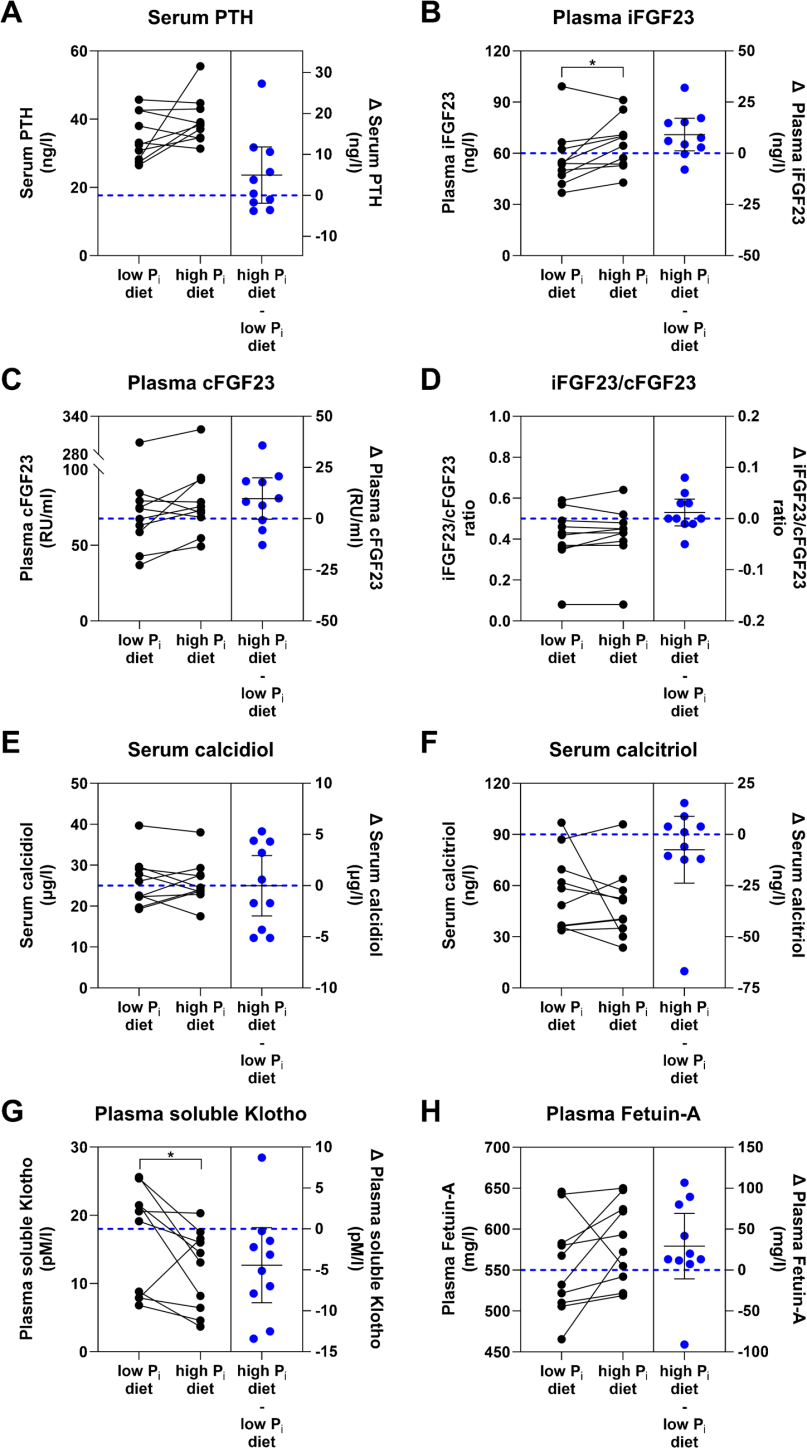
Phosphatropic hormones after 5 days of low- or high-phosphate (P_i_) diet. Levels of **A** serum parathyroid hormone (PTH), **B** plasma intact fibroblast growth factor 23 (iFGF23), **C** plasma C-terminal FGF23 (cFGF23), **D** the ratio of iFGF23 (ng/l)/cFGF23 (ng/l) as well as serum **E** calcidiol, and **F** calcitriol, and **G** plasma soluble Klotho, and **H** Fetuin-A on the final day of both the 5-day low and high P_i_ diet. One reference unit (RU)/ml corresponds to 2 ng/l cFGF23. Blue dots represent differences (Δ) between high P_i_ diet and low P_i_ diet expressed as mean difference ± 95% confidence interval and blue dashed line represents the zero line of the right y-axis. Data was analyzed by the paired *t*-test (**A**–**E**) or Wilcoxon test (**F–H**), *n* = 10, *p < 0.05

The study participants exhibited no significant difference in serum PTH between low and high dietary P_i_ intake on day 5 (Fig. 3A, Table 4), whereas high dietary P_i_ intake significantly elevated plasma iFGF23 but not cFGF23 (Fig. 3B and C, Table 4). The iFGF23/cFGF23 ratio was on average 0.4 however was not dependent on the diet (Fig. 3D). One study participant had a consistent and diet-independent much higher cFGF23 level compared to the other participants which also resulted in a strongly reduced iFGF23/cFGF23 ratio. The levels of both serum calcidiol and calcitriol remained unchanged at both time points (Fig. 3E and F, Table 4). High dietary P_i_ intake significantly reduced plasma soluble Klotho and tended to increase Fetuin-A levels (Fig. 3G and H, Table 4).

**Table 4:** Plasma/serum and urine hormones after 5 days of low- or high-phosphate (P_i_) diet. Data are presented either as mean ± SD (paired *t*-test) or median and interquartile range (Wilcoxon test)

Parameter	Low P_i_ diet Day 5 (*n* = 10)	High P_i_ diet Day 5 (*n* = 10)	*p*-value^1,2^
Plasma/serum			
Serum PTH (ng/l)	34.7 ± 7.0	39.7 ± 6.8	0.138^1^
Serum calcidiol (mg/l)	25.9 ± 6.1	25.8 ± 5.4	0.994^1^
Serum calcitriol (ng/l)	53.5 (36.1–73.9)	46.0 (33.8–58.9)	0.477^2^
Plasma iFGF23 (ng/l)	56.7 ± 17.3	65.8 ± 15.0	**0.029**^1^
Plasma cFGF23 (RU/ml)	88.0 ± 75.6	97.8 ± 79.1	0.056^1^
Plasma soluble Klotho (pM/l)	19.9 (7.9–22.5)	13.8 (6.0–16.8)	**0.049**^2^
Plasma Fetuin-A (mg/l)	549.9 (509–598)	582.7 (537–630)	0.061^2^
Serum aldosterone (ng/l)	63.6 ± 23.2	63.6 ± 22.0	0.998^1^
Serum epinephrine (nmmol/l)	0.15 (0.11–0.21)	0.14 (0.09–0.21)	0.813^2^
Serum norepinephrine (nmol/l)	0.72 ± 0.33	0.81 ± 0.20	0.400^1^
Serum dopamine (nnmol/l)	0.082 ± 0.060	0.082 ± 0.048	0.610^1^
Urine			
Metanephrine (ng/24 h)	139.8 ± 77.24	103.1 ± 60.5	0.226^1^
Dopamine (nmol/24 h)	1688 ± 365	1789 ± 788	0.711^1^

^1^Paired *t*-test, *p* < 0.05 are bold

^2^Wilcoxon test, *p* < 0.05 are bold

**Fig. 4:**
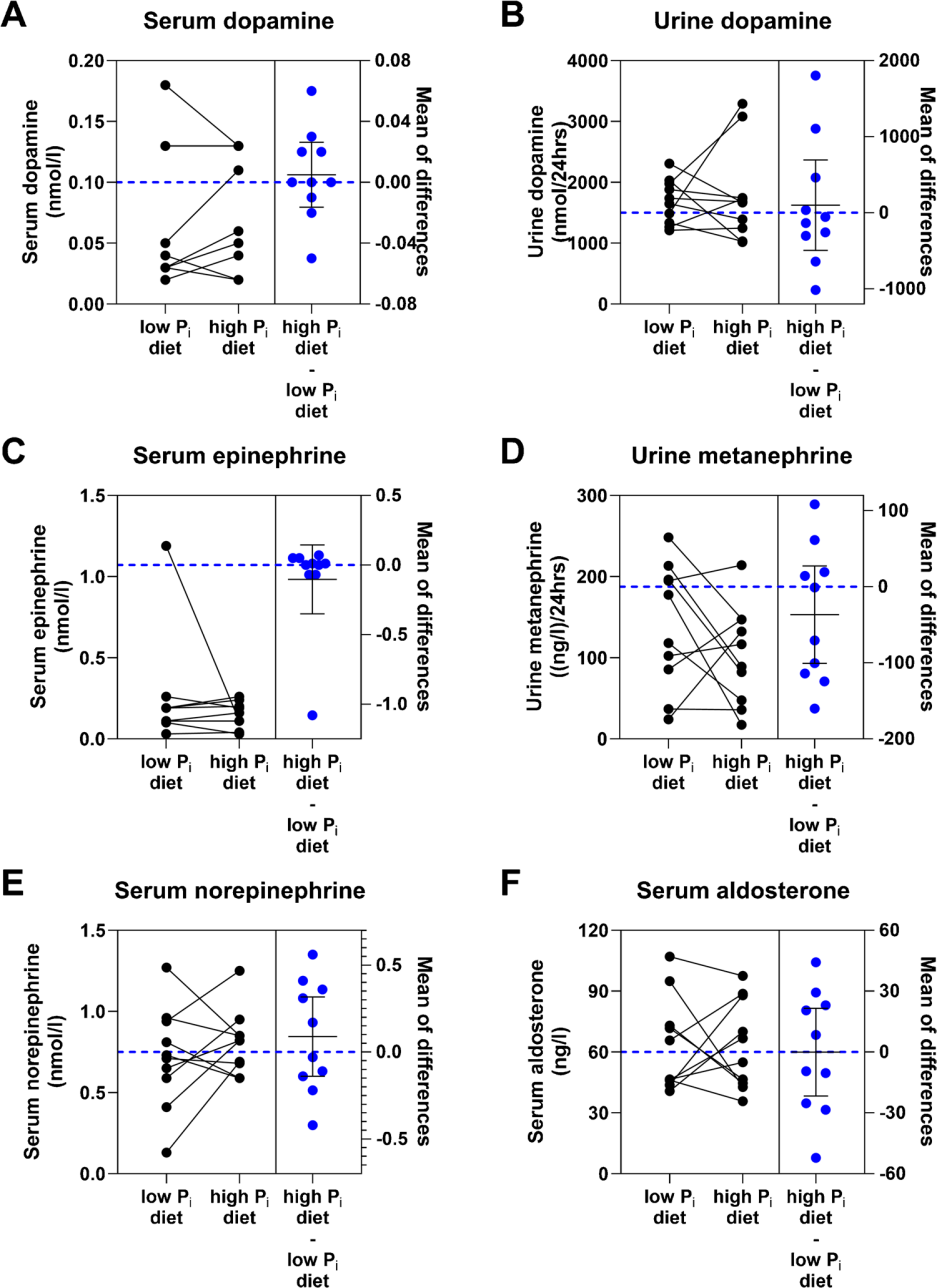
Serum and urine levels of hormones linked to mineral intake and control after 5 days of low- or high-phosphate (P_i_) diet. Serum and 24-h urine levels of **A**, **B** dopamine, **C** serum epinephrine, and **D** 24-h urine metanephrine, serum (**E**) norepinephrine, and (**F**) aldosterone. Blue dots represent differences (Δ) between high P_i_ diet and low P_i_ diet expressed as mean difference ± 95% confidence interval and blue dashed line represents the zero line of the right y-axis. Data was analyzed by the paired *t*-test (**A**–**B**, **D**–**F**) or the Wilcoxon test (**C**), *n* = 10

There were no diet-dependent differences in serum or 24-h urine excretion of dopamine, plasma epinephrine, norepinephrine, and aldosterone, and 24-h urine excretion of metanephrine (Fig. 4, Table 4).

**Fig. 5:**
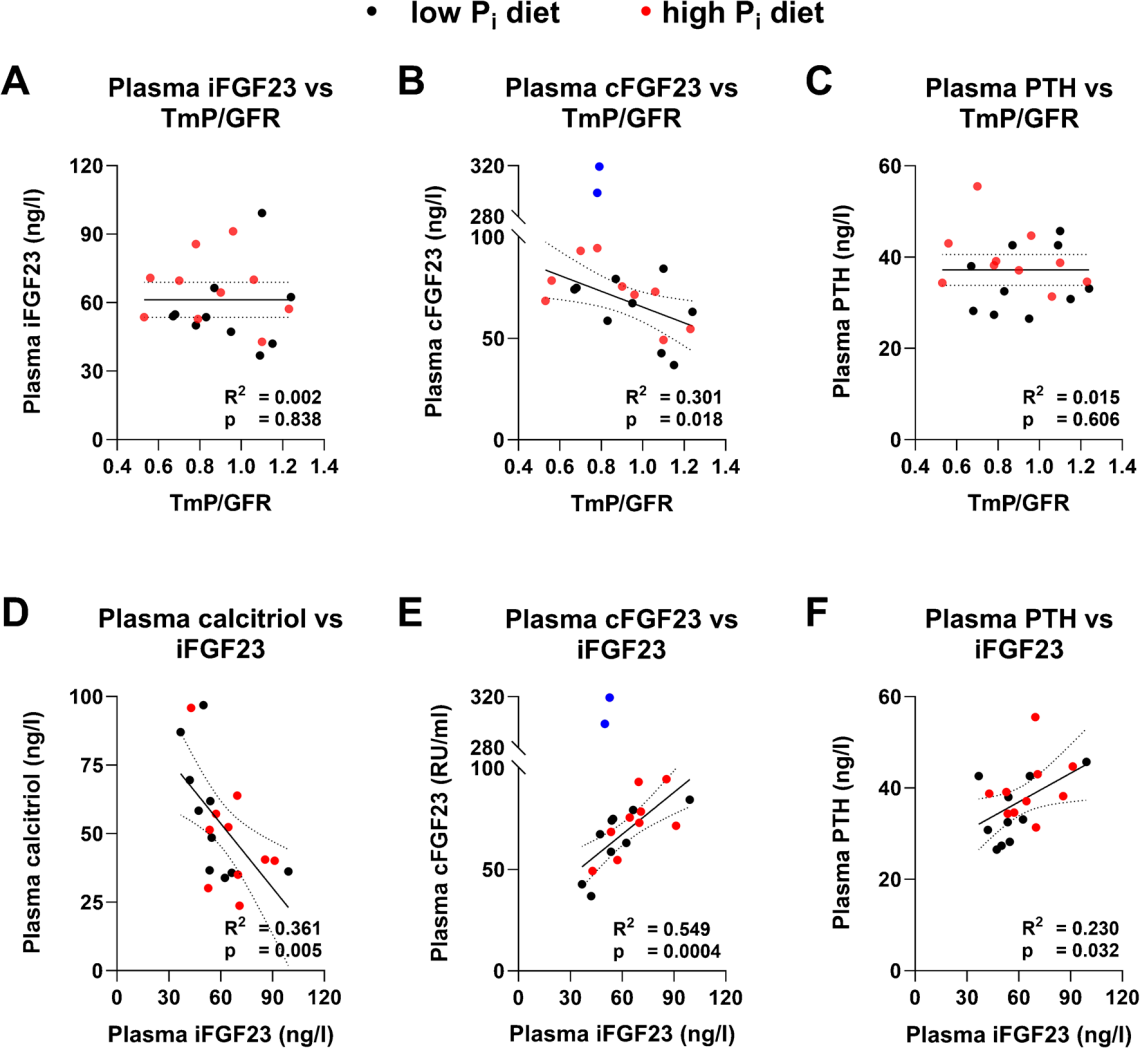
Associations between phosphatropic hormones and tubular maximal phosphate reabsorption. Non-linear regression analysis of plasma intact fibroblast growth factor 23 (iFGF23) (**A**), C-terminal FGF23 (cFGF23) (**B**), and serum parathyroid hormone (PTH) (**C**) with tubular maximum phosphate (P_i_) reabsorption (TmP/GFR) and of serum calcitriol (**D**), plasma cFGF23 (**E**), and serum PTH (**F**) with plasma iFGF23. A straight black line was fitted and ROUT method for outlier removal was applied. Black and red dots represent participants on low or high P_i_ diet, respectively. Blue dots represent identified outliers. The dashed line represents 95% confidence interval. The slope was analyzed by the extra sum-of-squares *F* test. Significance level *p* < 0.05

FGF23 and PTH are both potent negative regulators of P_i_ reabsorption in the kidney [32, 51]; however, non-linear regression analysis did not reveal any relationship between TmP/GFR and iFGF23 or PTH, respectively, whereas TmP/GFR and cFGF23 showed a significant negative relationship (Fig. 5A–C). Conversely, plasma iFGF23 had a significant negative relationship with calcitriol and a significant positive relationship with cFGF23 and PTH (Fig. 5D–F).

## Discussion

In this study, we examined the effects of a controlled 5-day low and high P_i_ diet on plasma minerals, endocrine regulators of phosphate metabolism, and renal mineral excretion in healthy young men. Our data demonstrate that under these controlled conditions, plasma P_i_, urinary P_i_ excretion, and daily FEP_i_ were significantly elevated when study participants were on high dietary P_i_ intake while TmP/GFR and daily renal filtered phosphate load were unchanged. These changes were accompanied by significantly elevated plasma iFGF23, reduced plasma soluble Klotho levels, and decreased urinary calcium excretion while all other factors related to phosphate homeostasis remained unchanged.

Only a few controlled studies in healthy subjects have addressed the impact of dietary changes in P_i_ intake on plasma P_i_ and endocrine changes. However, distinct differences exist compared to our study. Previous studies addressed either shorter (i.e., 8 h to 2 days) or longer (5 weeks) periods of standardized P_i_ intake, often combined with changes in calcium intake, and included women and men but without reporting sex specific data [25, 34, 41, 44, 50, 52, 58, 59]. Here we report data on only men with a well-defined age range and comparing the same subjects on a low and high P_i_ intake protocol using a cross-over design. All subjects had normal kidney function and no other known diseases.

Epidemiological data have shown associations between dietary P_i_ intake and serum P_i_ levels [22, 42, 49]. However, assessment of dietary P_i_ intake is difficult and imprecise due to the use of questionnaires, incomplete food composition tables, and the variable bioavailability of different types of phosphates (e.g., inorganic vs organic vs phytates). Only few studies in humans have directly assessed whether higher P_i_ intake would increase blood P_i_ levels, a question highly relevant as further association studies suggest that higher blood P_i_ levels are associated with higher all-cause and cardiovascular mortality and with lower bone mass [9, 15, 16, 25, 55]. While it is accepted that higher P_i_ intake can result in hyperphosphatemia in patients with impaired kidney function, the relationship is less clear in healthy subjects. Our data clearly show that an elevation of P_i_ intake over only 5 days is sufficient to significantly increase plasma P_i_ levels in subjects with normal kidney function. The data thus support the notion that higher P_i_ content in nutrients can cause higher plasma levels within the normal range. Whether these higher P_i_ levels are causative for higher cardiovascular morbidity and mortality remains still to be clarified. Data from animal studies and healthy subjects indicate that longer treatment with high P_i_ can lead to cardiac remodeling in animals and higher blood pressure in humans [19, 35, 42].

In our study, the average urinary excretion of P_i_ of 22.3 mmol/day (702 mg/day) and 41.2 mmol/day (1277 mg/day) reflects low and high dietary P_i_ intake and provides evidence that participants adhered to their diet. During the high dietary P_i_ intake period, study participants decreased urinary calcium excretion which may indicate interactions between P_i_ and calcium in the intestine causing precipitations of calcium phosphate that cannot be absorbed leading to lower urinary calcium excretion and limitation of intestinal phosphate absorption. Another possibility for the decrease in urinary calcium excretion may be an increased complexation of calcium with P_i_ due to increased plasma P_i_ levels and the subsequent formation of calciprotein monomers (CPM) and calciprotein particles (CPP) [53]. Of note, recommended daily allowance for phosphorus is 700 mg in adults and our low P_i_ diet thus reflects not a P_i_ replete condition but recommended dietary intake. In contrast, our high P_i_ diet rather reflects real P_i_ intake in many industrialized countries as suggested by data from the National Health and Nutrition Examination Survey (NHANES) showing average phosphorus intake in adults in the range of 1200–1400 mg/day [21]. Thus, both of our groups reflect recommended versus actual conditions and provide highly relevant information on the endocrine responses.

The endocrine response to changes in dietary P_i_ has been extensively studied in experimental animals, while less is known in healthy humans. Acute increases in dietary P_i_ intake as well as enteral or i.v. P_i_ loading cause a rapid increase in PTH and calcitriol, and timely delayed elevation of iFGF23 [44, 50, 59]. Two to five days of high dietary P_i_ intake increases iFGF23 but not cFGF23 [20, 25, 34, 58]. The sensitivity of the cFGF23 to indicate higher plasma P_i_ levels may be lower than for iFGF23 [17]. In addition, Ferrari and Vervloet found no changes in PTH most likely because both studies also adapted dietary calcium intake when changing P_i_. In the longest trial reported to date, Mohammad and colleagues found that 5 weeks of P_i_ supplements increased PTH, FGF23, and circulating Klotho levels while calcitriol was not altered [41]. Interestingly, also an increase in urinary metanephrine and normetanephrine was detected. Our data add to these observations and demonstrate that the relevant increase in P_i_ intake had no effect on PTH and calcitriol while it increased iFGF23 but not cFGF23. Despite the increase in iFGF23, there was surprisingly no association between iFGF23 and TmP/GFR but rather a negative relationship between cFGF23 and TmP/GFR. The association of cFGF23 with TmP/GFR cannot be explained by a change in iFGF23/cFGF23 as this ratio remained stable. The changes in serum P_i_ and plasma iFGF23 introduced by the high dietary P_i_ intake were minimal and the calculation of TmP/GFR at a single time point might not accurately represent minimal changes in iFGF23, while the phosphaturic hormones did not correlate with TmP/GFR they correlated according to their expected action with each other. Calcitriol showed a significant negative correlation with iFGF23 and lower calcitriol levels likely reflecting the suppression of calcitriol synthesizing enzyme CYP27B1 and stimulation of the catabolic CYP24A1 enzyme by iFGF23 as observed in animal studies [31, 33, 51]. Furthermore, PTH showed a positive correlation with iFGF23 suggesting that in the current study elevated iFGF23 did not inhibit PTH secretion but rather PTH stimulated iFGF23, eventually to lower plasma P_i_ [4, 39]. The correlations must be taken with caution as the measurements of study participants on each diet are dependent on each other. However, the low number of participants did not allow for more sophisticated analysis. In our study, we found that high dietary P_i_ intake reduced plasma soluble Klotho levels. This result is consistent with the finding from a small population-based study cohort, where serum soluble Klotho levels were negatively associated with the estimated dietary P_i_ intake [23]. Similarly, in rodent studies, renal Klotho expression—the main source of plasma soluble Klotho [38]—is upregulated and downregulated with low and high dietary P_i_, respectively [18, 40]. However, contrasting results were reported by Mohammed et al. who found an increase in soluble Klotho with high dietary P_i_ intake. This discrepancy might be due to the different study length or different assay method [41]. Further studies with more study participants are needed to confirm the downregulation of soluble Klotho by high dietary P_i_. Fetuin-A is an inhibitor of calcium phosphate precipitation in the blood [54] and has been shown to be not regulated in the post-prandial response of healthy volunteers [53]. In our study, plasma Fetuin-A levels slightly increased during the period of high P_i_ diet with one exemption which might indicate that an increase in P_i_ also increases the phosphate buffering system in the blood to avoid P_i_ precipitation. Further studies with more study participants and determination of CPPs are required to validate this effect. We did not find an increase in plasma epinephrine and norepinephrine nor in urinary metanephrine excretion. Several reasons may explain this discrepancy, such as the higher P_i_ supplementation or the longer treatment period used by Mohammad et al. [41].

Last we measured also other hormones that have been linked to phosphate homeostasis such as dopamine and aldosterone [3, 10, 12, 13, 24, 47, 57, 61, 62]. Dopamine was measured both in plasma and urine while aldosterone only in urine. However, we did not detect any changes in these hormones. We speculate that they are either altered with different kinetics and that we missed their regulation, that dopamine may only be changed locally in the kidney, or that their regulation requires more pronounced differences in dietary P_i_ intake.

One limitation of this study is the unequal sodium intake between the P_i_ diets. Participants had inadvertently 840 mg higher sodium intake when receiving the low P_i_ diet compared to the high P_i_ diet. The literature suggests that high sodium intake is associated with lower cFGF23 expression [27, 29, 48, 62]. However, these studies either used extremely high sodium supplementation (18 g NaCl) or are in addition associated with lower eGFR, or hyponatremic patients were studied. In our study the difference in supplementation is limited to 840 mg, eGFR is not affected by the intervention, and we nevertheless observed an increase in iFGF23 but not cFGF23. Therefore, the difference in iFGF23 and P_i_ levels likely originates only from the difference in P_i_ rather than sodium intake.

In summary, we performed a controlled cross-over trial in healthy young men with two different levels of dietary P_i_ intake corresponding to either the recommended daily allowance or to current levels of “real-world” P_i_ intake in the general population. Our data demonstrates that higher P_i_ intake increased plasma P_i_, iFGF23 levels, and daily FEP_i_, and decreased urinary calcium excretion and plasma soluble Klotho. Plasma iFGF23 also correlated with lower calcitriol and higher PTH levels while other hormones linked to phosphate such as dopamine, (nor)epinephrine, and aldosterone showed no regulation under the conditions studied. Our data provide experimental evidence that higher dietary P_i_ intake increases plasma P_i_ levels even in subjects with normal kidney function as observed in association studies and that this increase might have implications for associated cardiovascular disease.

## Supplementary Information

Below is the link to the electronic supplementary material.

Supplementary file1 (PDF 207 KB)

## Data Availability

The data underlying this article cannot be shared publicly for the privacy of individuals that participated in the study*.* Anonymized data will be shared upon reasonable request to the corresponding author.

## Footnotes

1 There is no elemental phosphorus in the body or diet, just organic and inorganic phosphate. However, the laboratory measures and reports phosphorus content. Phosphorus and phosphate are equivalent in millimoles but not in milligrams. The term P_i_ is used broadly to denote the mass in milligrams of phosphorus that is present in the organic and inorganic phosphate. It is strictly speaking not correct but it is the conventional laboratory parlance. P_i_ in this paper refers to phosphorus mass content in all species of phosphate.
