# Supplementary material for: Controlled dietary phosphate loading in healthy young men elevates plasma phosphate and FGF23 levels
Source: Pflugers Arch. 2024 Nov 27;477(3):495–508. doi: 10.1007/s00424-024-03046-4 (PMC11825603; doi:10.1007/s00424-024-03046-4)
Supplement: Supplementary file 1 — Supplementary file1 (PDF 207 KB) [file 424_2024_3046_MOESM1_ESM.pdf]

Nutrition guidelines phosphate loading study

Breakfast:

- Individually at home
- Bread: Whole wheat bread, Semmeli, St. Galler white bread (no bun, braid, or lay pastries)
- Butter (regular)
- Jam or honey
- Camembert: 30g
- Fruit yogurt/quark: 125 g ready-made product
- Yogurt: Not allowed if it contains nuts, chocolate, or Birchermuesli

Morning snack

- Fruit (self-chosen) 1 portion (approx. 120 g)

Lunch

- From 11:30 am to 2:00 pm at the staff restaurant Fantasia: State your name
- Dinner can be taken as lunch from the staff restaurant Fantasia (if desired, it must be ordered the day before by 2:00 pm via phone: 51850/1)
- Menu served in microwaveable takeaway containers

Afternoon snack

- Participant receives fruit to take along or can get their own (fruit) 1 portion

Dinner

- From 4:00 pm to 7:00 pm at Bistro Süd
- Menu served in microwaveable takeaway containers

Drinks

Allowed:	Water, tea (unsweetened or sweetened), soft drinks (except cola drinks of all brands), Rivella (and other whey-based drinks), coffee (up to 1 liter without milk, sugar
Forbidden:	All other drinks, fruit juices, vegetable juices, milk, yogurt drinks, Ovaltine, chocolate drinks, etc.
Alcohol:	2 dl of white wine or 2 dl of red wine or 2 dl of beer (can be diluted with lemonade) per day is allowed

In case of hunger:

- Sorbet: an additional 100 g and/or gummy bears: an additional 50 g
- If everything is exhausted: Mailaenderli or Chraebeli: an additional 30 g

UniversitätsSpital
Zürich

Klinik für Endokrinologie, Diabetologie
& Klinische Ernährung
Ernährungsberatung

Menu phosphate loading study

Study day	Breakfast	Lunch	Dinner
1	2 slices of house bread<br>2 butters<br>Jam<br>1 egg<br>Snack: fruit	Sliced beef<br>Dry rice<br>Tomato<br>Caramel cream	Fried veal schnitzel<br>Pasta<br>Vegetables<br>Fruit
2	2 slices of house bread<br>2 butters<br>Jam<br>Fruit yogurt, 150 g<br>Snack: fruit	Sliced veal<br>Polenta<br>Kohlrabi<br>Fruit cream	Fried chicken breast<br>Sauce<br>Roast potatoes<br>Broccoli<br>Fruit
3	2 slices of house bread<br>2 butters<br>Jam<br>Camembert, 30 g<br>Snack: fruit	Fried trout fillet<br>Risotto<br>Leek<br>Mixed leaf salad<br>Pineapple	Sliced chicken<br>Pasta<br>Carrots<br>Fruit
4	2 slices of house bread<br>2 butters<br>Jam<br>Camembert, 30 g<br>Snack: fruit	Veal schnitzel<br>Meat sauce<br>Spaetzli<br>Celery<br>Mixed leaf salad<br>Pear compote	Tofu ragout with vegetables<br>Couscous<br>Fruit or cookies
5	2 slices of house bread<br>2 butters<br>Jam<br>Fruit quark, 125 g<br>Snack: fruit	White veal stew<br>Pasta<br>Peppers<br>Raspberry cream puff	Vegetable ragout with veal<br>Dry rice<br>Apple
6 (washout)	2 slices of house bread<br>2 butters<br>Jam<br>Fruit quark, 125 g<br>Snack: fruit	Fried salmon<br>Boiled potatoes<br>Zucchini<br>Baked apple with vanilla cream	Apple tart<br>Bread<br>Butter
7 (washout)	2 slices of house bread<br>2 butters<br>Jam<br>Camembert, 30 g<br>Snack: fruit	Veal meatloaf<br>Sauce<br>Polenta<br>Beans<br>Peach with vanilla cream	Café complet (camembert,<br>cottage cheese<br>butter)<br>Fruit
8 (washout)	2 slices of house bread<br>2 butters<br>Jam<br>1 egg<br>Snack: fruit	Sliced beef<br>Dry rice<br>Tomato<br>Caramel cream	Fried veal schnitzel<br>Pasta<br>Vegetables<br>Fruit

9 (washout)	2 slices of house bread<br>2 butters<br>Jam<br>Fruit yogurt, 150 g<br>Snack: fruit	Sliced veal<br>Polenta<br>Kohlrabi<br>Fruit cream	Fried chicken breast<br>Sauce<br>Roast potatoes<br>Broccoli<br>Fruit
10 (washout)	2 slices of house bread<br>2 butters<br>Jam<br>Camembert, 30 g<br>Snack: fruit	Fried trout fillet<br>Risotto<br>Leek<br>Mixed leaf salad<br>pineapple	Sliced chicken<br>Pasta<br>Carrots<br>Fruit
11 (washout)	2 slices of house bread<br>2 butters<br>Jam<br>Camembert, 30 g<br>Snack: fruit	Veal schnitzel<br>Meat sauce<br>Spaetzli<br>Celery<br>Mixed leaf salad<br>Pear compote	Tofu ragout with vegetables<br>Couscous<br>Fruit or cookies
12 (washout)	2 slices of house bread<br>2 butters<br>Jam<br>Fruit quark, 125 g<br>Snack: fruit	White veal stew<br>Pasta<br>Peppers<br>Raspberry cream puff	Vegetable ragout with veal<br>Dry rice<br>Apple
13	2 slices of house bread<br>2 butters<br>Jam<br>1 egg<br>Snack: fruit	Sliced beef<br>Dry rice<br>Tomato<br>Caramel cream	Fried veal schnitzel<br>Pasta<br>Vegetables<br>Fruit
14	2 slices of house bread<br>2 butters<br>Jam<br>Fruit yogurt, 150 g<br>Snack: fruit	Sliced veal<br>Polenta<br>Kohlrabi<br>Fruit cream	Fried chicken breast<br>Sauce<br>Roast potatoes<br>Broccoli<br>Fruit
15	2 slices of house bread<br>2 butters<br>Jam<br>Camembert, 30 g<br>Snack: fruit	Fried trout fillet<br>Risotto<br>Leek<br>Mixed leaf salad<br>Pineapple	Sliced chicken<br>Pasta<br>Carrots<br>Fruit
16	2 slices of house bread<br>2 butters<br>Jam<br>Camembert, 30 g<br>Snack: fruit	Veal schnitzel<br>Meat sauce<br>Spaetzli<br>Celery<br>Mixed leaf salad<br>Pear compote	Tofu ragout with vegetables<br>Couscous<br>Fruit or cookies
17	2 slices of house bread<br>2 butters<br>Jam<br>Fruit quark, 125 g<br>Snack: fruit	White veal stew<br>Pasta<br>Peppers<br>Raspberry cream puff	Vegetable ragout with veal<br>Dry rice<br>Apple
